# The Earlier You Find, the Better You Treat: Red Flags for Early Diagnosis of Inflammatory Bowel Disease

**DOI:** 10.3390/diagnostics13203183

**Published:** 2023-10-12

**Authors:** Laura Cantoro, Rita Monterubbianesi, Giuliano Falasco, Caterina Camastra, Paolo Pantanella, Mariangela Allocca, Rocco Cosintino, Roberto Faggiani, Silvio Danese, Gionata Fiorino

**Affiliations:** 1 IBD Unit, Department of Gastroenterology and Digestive Endoscopy, San Camillo-Forlanini Hospital, 00152 Rome, Italy; lcantoro@scamilloforlanini.rm.it (L.C.); gfalasco@scamilloforlanini.rm.it (G.F.);; 2 Department of Gastroenterology and Digestive Endoscopy and Vita-Salute San Raffaele University, 20132 Milan, Italy; allocca.mariangela@hsr.it (M.A.); danese.silvio@hsr.it (S.D.)

**Keywords:** diagnostic delay, Crohn’s disease, ulcerative colitis, inflammatory bowel disease, red flags, calprotectin

## Abstract

Delayed diagnosis is a challenge in the management of inflammatory bowel disease (IBD). Several studies show a significant association between diagnostic delay and disease progression to complications and surgery, especially in Crohn’s disease (CD). What risk factors are associated with diagnostic delay in IBD remains unclear. In order to reduce diagnostic delay, the Red Flags Index has been developed and validated. The combination of the Red Flags Index score and non-invasive biomarkers such as fecal calprotectin seems to be highly accurate in screening patients with underlying IBD to be referred for further diagnostic workup and eventual early effective treatment strategies. Our literature review aims to obtain a comprehensive overview of the impacts of diagnostic delay in IBD on the potential risk factors associated with IBD, how diagnostic tools may be effective in reducing diagnostic delay, and future perspectives in this field.

## 1. Introduction

Inflammatory bowel diseases (IBDs) are a group of immune-mediated diseases, namely, Crohn’s disease (CD) and ulcerative colitis (UC), which share common pathogenetic mechanisms and similar clinical aspects. While UC only affects the colonic mucosa, CD can extend to any segment of the gastrointestinal tract.

The persistence or recurrence of inflammation can result in progressive damage to the gastrointestinal tract, potentially resulting in strictures, penetrating disease, abscesses [1], and even dysplasia and cancer [2]. These complications result in 50% and 15% of individuals with CD and UC, respectively, requiring surgery within 10 years of diagnosis [3].

Delayed diagnosis of IBD may potentially impact disease progression and subsequent clinical outcomes [4,5,6,7]. Poor clinical outcomes may also negatively impact psychological well-being, quality of life, and work productivity, at a considerable cost to the individual and the economy [4,5,6,7].

## 2. IBD Pathogenesis

**Figure 1:**
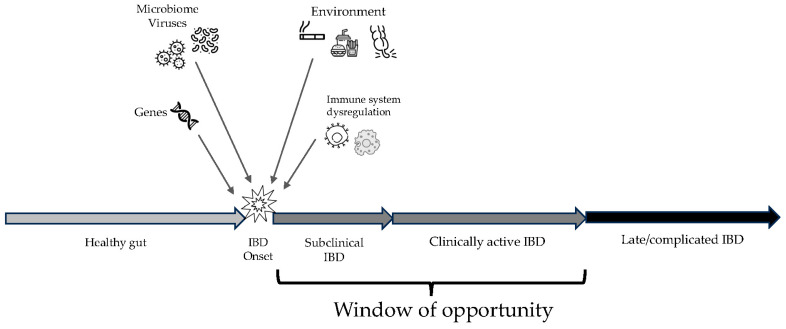
Overview on the progression of inflammatory bowel disease (IBD), from the onset of inflammation to late complications. The window of opportunity includes the best time frame to enhance effectiveness of therapies.

IBDs share common pathogenetic mechanisms, although their etiology still remains unknown [8]. In both CD and UC, although some mechanisms are different, it seems that the complex interaction between genetic predisposition, the innate and adaptive immune systems, the microbiome, and environmental factors could be the trigger to initiate and then maintain active chronic inflammation [8,9,10] (Figure 1). Some genes seem to be associated with an increased risk of IBD, such as NOD2, ATG16L1 T300A, CARD9, TNFSF15/TL1A, IL-23R, and IL-10, although their presence alone may be not sufficient to trigger IBD [11]. The role of the microbiome still remains to be elucidated, although it seems that increased permeability of the gut epithelium and loss of tolerance for the host microbiota by the immune system may play a key role in triggering and maintaining inflammation [12]. More recent studies suggest that the contact between certain eukaryotic viruses might trigger intestinal inflammation and contribute to IBD pathogenesis [13]. Environmental factors, such as diet, smoking, and appendectomy, may also have an impact on IBD pathogenesis, although they can impact differently in CD and UC [14].

## 3. Diagnostic Delay in IBD Population

When chronic inflammation starts in the affected gut, this process may take time to result in clinically significant manifestations of IBD. Therefore, there could be a time lag between the onset of inflammation itself and the onset of symptoms and signs that could lead to a proper and timely diagnosis. This subclinical inflammation is actually hard to find and treat and can sometimes lead to bowel damage even before becoming clinically active [15]. If clinically active inflammation is diagnosed before evolving into complications, there is the possibility to manage and treat the disease within the so-called window of opportunity, when the probability of success of any intervention is the highest possible [1,16].

Although the usual symptoms of IBD are chronic diarrhea, abdominal pain, weight loss, and rectal bleeding, up to 30% of patients with IBD have silent disease [17,18]. This makes the diagnosis of inflammatory bowel disease (IBD) challenging. On the other hand, the limited knowledge of IBD among the general population and non-specialist gastroenterologists or general practitioners (GPs) may lead to misunderstanding the symptoms and signs of underlying IBD, resulting in a significant delay in diagnosing and referring patients with IBD [17,18].

Timely diagnosis and referral for the first therapeutic approach allow one to obtain all the advantages of being treated within the window of opportunity [16]. Some studies report the median duration of symptoms before diagnosis to be 2–12 months for CD and 2–7 months for UC [3,11,12,13,14]. The IMPACT survey conducted on 5576 patients with IBD, however, reported that approximately 48% of patients needed to wait more than one year to obtain an established diagnosis of IBD, and approximately 25% of patients had to wait up to 5 years from the onset of symptoms to obtain a final diagnosis of IBD, despite being seen by a doctor or despite having one or more visits to the emergency room [15]. The length of diagnostic delay appears to not have changed across six decades (1955–2014), as reported by Cantoro et al., while a progressive reduction in the proportion of patients with a long diagnostic delay (longer than 24 months) over the last three decades has been observed. A recent review also found that the extent of delay remained relatively consistent over time from 2009 to 2021 for IBD and UC. However, for the same period, studies focused on CD have shown greater fluctuations year-on-year in the reported extent of delay [14]. Few studies have reported on the relative contributions of patients- and healthcare-related intervals to the overall time to diagnosis. Vavricka et al. found that for CD patients, the time interval from the first outpatient clinic evaluation to IBD diagnosis was much longer (median 4 months) than the time interval from the onset of symptoms to the first access to a specialist (median 2 months), leaving room for improvement. In contrast with CD, the time intervals from physician visits to UC diagnoses are comparable to the time intervals from first symptoms to physician consultations. Cantoro et al. also found that IBD patient-dependent diagnostic delay, i.e., the time between the onset of symptoms and the first medical consultation, was significantly higher than physician-dependent diagnostic delay, i.e., the time between the first medical consultation and final diagnosis [11]. A possible explanation for this difference could be the quite high prevalence of IBS-like symptoms at IBD onset, which may be underestimated by the patient himself/herself. Another possible reason could be the difficulty that patients may have in accessing healthcare systems in different countries. This aspect seems to be important when we compare the diagnostic delay between countries, as in some countries, such delay is hugely different from and longer than in other ones (Table 1) [19]. In the sub-analysis of one single cohort out of the entire study population, Cantoro et al. [20] showed that 163 patients (29%) had at least one misdiagnosis, and this was significantly (*p* = 0.006) more frequent in CD (68%) than in UC (32%). For both diseases, the most frequent misdiagnosis was IBS (36.0% for CD and 38.5% for UC). In contrast, Jayasooriya et al. indicated that the median of the healthcare-related interval to overall diagnosis was longer than the patient-related interval in CD [19]. Meanwhile, the median of the patient-related interval was found to be longer among patients diagnosed with UC [13]. Most of the studies do not account for the baseline prevalence of GI symptoms in the general population and may overestimate the duration and prevalence of symptoms attributable to undiagnosed IBD.

Blackwell et al. examined the prevalence of GI symptoms in the decade before IBD diagnosis compared with matched control groups drawn from the general population [17]. They reported a significant excess of GI symptoms amongst patients who go on to develop IBD in each of the 10 years prior to their diagnoses compared with matched controls. The authors found one in ten individuals visited their primary care physician for GI symptoms 5 years before being diagnosed with either CD or UC, compared with one in twenty individuals in the matched control group. Only 5.6% of individuals with IBD received timely specialist review within 4 weeks of presenting to their primary care physician with chronic GI symptoms, although this rose to 15.2% in the most recent era of their study (2014–2016), perhaps reflecting improved diagnostic pathways with the increased use of surrogate biomarkers [17].

All these data show that diagnostic delay is a global challenge in IBD, as this impacts a large number of patients, irrespective of the country and the healthcare system.

## 4. Prevention of Negative Course of CD

Timely diagnosis and referral for the first therapeutic approach allow one to obtain all the advantages of being treated within the window of opportunity. Data from population-based cohort studies show that early non-complicated CD may evolve into complications within 90 days, with a cumulative risk of 18.6%, suggesting that early intervention is crucial in changing the natural history of the disease. Although early intervention with therapy is suggested for high-risk CD patients, a consensual definition of early CD is still lacking. To address this issue, an international panel of experts in IBD developed a clear definition of early CD. Hence, the Paris criteria defining early CD include (1) a disease duration of <18 months and (2) no previous or current use of disease-modifying agents [23]. Data from randomized clinical trials with immunosuppressants and biologics suggest that treating patients with a disease duration of <2 years and an absence of complications may significantly reduce the risk for complications and increase the time in remission in patients with CD. Moreover, rapid disease control may effectively prevent disease progression and allow dose reduction or even the withdrawal of treatment, reducing the risk of long-term adverse events and healthcare costs. Recent data from pivotal trials in patients with CD suggest that anti-TNF therapy is more effective in early disease. Two main reasons may contribute to the greater efficacy of anti-TNF therapy in early CD: immunology and the high percentage of patients with pure inflammatory, uncomplicated disease [10].

Some studies report the median duration of symptoms before diagnosis to be 2–12 months for CD and 2–7 months for UC [3,19,20,24,25]. The IMPACT survey conducted on 5576 patients with IBD, however, reported that approximately 25% of patients had to wait up to 5 years from the onset of symptoms to obtain a final diagnosis of IBD, despite being seen by a doctor or despite having one or more visits to the emergency room [26].

Strategies to reduce diagnostic delay remain worthy of investigation.

## 5. Impact of Diagnostic Delay on Disease Outcome

The majority of studies report a significantly increased risk of complications and bowel damage associated with diagnostic delay. Schoepfer et al. conducted the first analysis of the impact of diagnostic delay on CD outcomes in a population of 905 patients and found that diagnostic delay was significantly associated with the occurrence of bowel stenosis (odds ratio (OR) 1.551, *p* = 0.047 for a delay of 4–9 months; OR 1.756, *p* = 0.011 for a delay of ≥25 months) and intestinal surgery (OR 1.495, *p* = 0.097 for a delay of 4–9 months; OR 1.757, *p* = 0.014 for a delay of 10–24 months; and OR 2.025, *p* = 0.003 for a delay of ≥25 months) [7]. Pellino et al. and Li et al. also found a significant risk of surgery in CD patients with delayed diagnosis [5,6]. The Korean cohort, including 165 CD and 130 UC patients, also demonstrated that a long diagnostic delay in patients with UC was an independent risk factor for intestinal surgery [5,6].

A Swiss IBD cohort study found a significantly higher prevalence and risk of bowel stenosis, internal fistulas, and any complication in the adult-onset CD population compared with the pediatric population. In the long term, the length of diagnostic delay was significantly associated with bowel stenosis, internal fistulas, and any complication in the adult-onset CD population, and no significant association between the length of diagnostic delay and CD-related outcomes was found in the pediatric population [16]. The same findings resulted from a retrospective cohort in South Korea. In that study, a long diagnostic delay (greater than 18 months) was independently predictive of the further development of intestinal stenosis, internal fistulas, and perianal fistulas [17]. Cantoro et al. showed a significantly higher percentage of patients with a complicated disease (36%) in comparison with those with an uncomplicated pattern of disease (25%) among CD patients with a long diagnostic delay (greater than 24 months) [11]. The US cohort showed that 63% of patients with CD after 26 months of symptom onset had at least one complication at the time of diagnosis compared with 25% of patients diagnosed within 4 months of symptom onset. Multivariable analysis showed increased odds of developing any complication at the time of diagnosis in patients with a longer time to diagnosis [19].

A recent metanalysis of 101 studies including 112,194 IBD patients found that delayed diagnosis was associated with higher odds of stricturing (OR = 1.88; CI: 1.35–2.62), penetrating disease (OR = 1.64; CI: 1.21–2.20), and intestinal surgery (OR = 2.24; CI: 1.57–3.19) in CD, whereas it was associated with higher odds of colectomy (OR = 4.13; CI: 1.04–16.40) in UC [13].

## 6. Risk Factors for Diagnostic Delay

Identifying predictors of diagnostic delay is an important issue when managing chronic diseases that have a high risk of disability. Some important risk factors associated with diagnostic delay in IBD have been identified, especially in CD. These factors vary across studies and, in some cases, even result in conflict. This is likely due to the different study populations examined, differences in disease behavior, healthcare settings, and the countries in which the studies were conducted [13,14].

Jayasooriya found the time to diagnosis was longer in studies from low- and middle-income countries compared with those from high-income countries, which may relate to differences in healthcare provision. Furthermore, difficulty in differentiating between IBD and more prevalent infectious diseases in those areas may increase the challenge of appropriate timely diagnosis of IBD in low- and middle-income countries [19].

In a Swiss IBD cohort, a multivariable analysis found that an age of <40 years (*p* = 0.039) and ileal location (*p* = 0.013) were significantly associated with diagnostic delay. The authors explained this finding by the fact that ileal location is more frequently revealed by abdominal pain only without diarrhea, which can be confused with irritable bowel syndrome (IBS) [3]. There was an association between the use of NSAIDs (OR 1.75; *p* = 0.094) and female gender (1.69; *p* = 0.079) and diagnostic delay in UC, but no factors were found to be related to this in the multivariable analysis.

Li et al. found a significant statistical difference between two groups of CD patients, CD diagnostic delay patients and non-diagnostic delay patients, regarding an age of more than 40 years at diagnosis (35.3% vs. 18.2%), basic educational level (48.2% vs. 30.6%), and no family history of Crohn’s disease (0 vs. 1.6%). Moreover, an interesting report from a Korean study provides evidence that perianal discomfort is significantly associated with long diagnostic delays, and the time from the first visit to the diagnosis (physician-dependent delay) was significantly longer in patients with perianal discomfort, although there was no difference in the time from symptom onset to the first hospital visit (patient-dependent delay). The authors considered this complaint to be related to the tendency of CD patients with perianal discomfort to visit colorectal/anus surgery clinics or general doctor’s clinics for the first time in South Korea and the tendency of those physicians to overlook and miss the diagnosis of CD because of anal disorders, such as hemorrhoids.

Nahon et al. investigated whether age at diagnosis, gender, a family history of IBD, CD location and behavior, a past history of appendectomy, extra-intestinal manifestations, the year of diagnosis, marital status, education, and a linguistic understanding of socioeconomic deprivation evaluated with a validated score (EPICES score) were associated with diagnostic delay. None of them impacted either diagnostic delay or the severity of CD [27]. Similar findings were reported in a cohort of patients from the United States, as an age of more than 40, sex, smoking status, non-steroidal anti-inflammatory drugs (NSAIDs) use, a family history of IBD, previous abdominal surgery, the presence of extra-intestinal manifestations (EIMs), and whether the diagnosis was made during a hospital admission did not seem to be associated with a delay in diagnostic time for patients with CD [28]. In this cohort, exclusive ileal involvement was confirmed to be associated with a longer time to diagnosis compared with patients without ileal involvement.

In one study from Italy, an age at diagnosis of >40 years and a complicated disease defined by a B2 + B3 phenotype were independently associated with a diagnostic delay of >24 months in CD patients. In UC patients, only an age at diagnosis of >40 years was associated with a diagnostic delay of >24 months.

Similar evidence came from another Italian cohort, where CD patients who received a diagnosis of penetrating disease showed a longer SDI (symptoms interval diagnosis) compared with other disease behaviors (*p* = 0.003). The risk of a delayed diagnosis was 21% for an inflammatory pattern, 27% for a stricturing pattern, and 59% for a penetrating pattern (OR penetrating vs. other patterns = 5.12) [6].

Eventually, the ileal localization of the disease, complicated behavior at diagnosis, and perianal discomfort appear to be the more frequent risk factors associated with diagnostic delay in CD. Age at diagnosis of CD appears to influence the time to diagnosis but the data are conflicting. Socioeconomic status does not seem to influence the time to diagnosis.

As highlighted by several studies, diagnostic delay is strongly and directly associated with negative outcomes and poor prognosis. This is the main reason why strategies directed at reducing delay are urgently needed.

## 7. Reducing Diagnostic Delay: The Red Flags Index

The main causes of diagnostic delay are yet to be clarified. Timely access to IBD units or any healthcare facility to diagnose and manage patients with suspicion of IBD is supposed to be the main challenge to face in order to reduce diagnostic delay. Fiorino et al. reported the rate of bowel damage in 142 newly diagnosed CD patients referred to two large referral centers in Italy and France. They found that less than 5% of patients were diagnosed in those two centers, whereas the rest of the patients were diagnosed elsewhere and then referred. This means that IBD diagnosis is very often not performed in IBD referral centers. In the overall study population (*n* = 142), 39.3% of patients had bowel damage at diagnosis assessed using any cross-sectional imaging technique [15] and scored using the Lémann Index [29,30]. If we try to estimate the diagnostic delay in this cohort based on the main studies on CD natural history by Cosnes et al., this cohort shows that 40% of patients with complications usually have a ≥3-year-old disease [23].

In order to help non-expert physicians, such as GPs or non-IBD-dedicated specialists, an IOIBD (International Organization for the Study of Inflammatory Bowel Diseases) initiative developed a red flag index for the early diagnosis of CD in patients presenting with GI symptoms [31]. Firstly, a 21-item questionnaire developed by worldwide experts in IBD was administered to 36 healthy subjects, 80 patients with irritable bowel syndrome (non-CD group), and 85 patients with recently diagnosed (<18 months) CD in three international IBD referral centers. This questionnaire allowed them to assess differences in the symptoms and signs of early CD compared with IBS and healthy status. Multiple logistic regression analyses selected and weighted independent items, which were used to construct the Red Flags Index. This index resulted in eight simple questions answered yes or no, which resulted in a final score, after multiplying by coefficients calculated via the multivariable analysis (Table 2). The ROC curve analysis showed that a score equal to or higher than eight was the best cut-off to discriminate early CD patients. Patients with a Red Flags Index score of ≥ 8 have a high probability of having underlying CD (OR 290; 95% CI 77–1086; *p* < 0.0001).

A validation study among GPs was conducted prospectively in Italy. From November 2016 to November 2019, 112 patients were included by 64 GPs in the region of Lombardy, Italy. All enrolled patients were scored with the Red Flags Index, and irrespective of the score, they were all referred to an IBD specialist for their first evaluation, diagnostic workup, and final visit. A total of 66 subjects (59%) completed the study after the first gastroenterological visit. The prevalence of CD was 3.6% in the study population (4/112), of which 75% (3/4) had non-stricturing non-penetrating active CD. The RF index had 50% sensitivity, 58% specificity, 4% PPV, and 97% NPV. A combined diagnostic strategy with fecal calprotectin (FC) (RFI ≥ 8 and/or FC > 250 ng/g) resulted in significantly improved accuracy: 100% sensitivity (29–100%), 72% specificity (55–85%), PPV = 21% (5–51%), and NPV = 100% (88–100%) [32].

The study confirmed that early identification of suspected CD resulted in higher chances of finding the disease within the correct window of opportunity to be effectively treated. On the other hand, the validation study confirmed that screening for CD based only on symptoms and signs can miss one-third of diagnoses, confirming the high number of patients with silent CD. This study also highlighted another challenge in the context of diagnostic delay. Although the validation study offered the opportunity to have easy and quick access to healthcare facilities as well as to IBD specialists free of charge, only 59% of patients completed the diagnostic workup. These findings suggest that timely referral is probably not the only challenge. Many patients may delay diagnosis by underestimating the importance of seeing a doctor and undergoing the prescribed diagnostic procedures. This may be related to the scarce knowledge and awareness of IBD in the general population, which might impact the diagnostic delay, too.

## 8. Discussion

Diagnostic delay is a very important challenge in IBD management. The evidence clearly shows that diagnostic delay, especially in CD, is associated with a higher risk of complications and major surgery. Population-based studies suggest that evolution to complications and bowel damage may occur even earlier than expected, as one in five patients may evolve from having a non-stricturing non-penetrating disease into any complication within 90 days and 1 year [33]. Early diagnosis is likely to allow timely early treatment with effective treatments. As for rheumatoid arthritis, IBD also shows a window of opportunity [1] where patients can have the highest chance to respond to therapies and limit the risk of disease evolution to complications, organ damage, and surgery [16]. In this perspective, the Red Flags Index and non-invasive first-line diagnostic tools such as fecal calprotectin might help in identifying patients with early-onset IBD who may benefit from prompt diagnoses and early referrals to IBD specialists. The timely management of IBD will result not only in a benefit to patients but also a significantly lower cost for healthcare systems [28]. Healthcare professionals who are not specialists in IBD or not gastroenterologists should be aware of the initial symptoms and signs of IBD (including those related to extra-intestinal manifestations) and, whenever it is possible, have a close connection with specialists in order to start the proper diagnostic workup as quickly as possible.

On the other hand, optimizing patients’ referrals may not be the only way to reduce or even remove diagnostic delays. Diagnostic delay also occurs in countries where healthcare access is easy for everyone. Therefore, awareness of IBD in the general population may also be part of this challenge. Although there are several differences between the symptoms and signs of CD and irritable bowel syndrome, which is very common in the general population, the overlap of symptoms between the two conditions and the relapsing–remitting behavior of symptoms in CD may mislead a patient, delaying or even avoiding being seen by a doctor.

Another point to be considered is the absence of red flags that may occur, limited to symptoms in up to 30% of patients [34]. This was also the problem of the relatively low sensitivity of the Red Flags questionnaire, as it was mainly based on symptoms. Its combination with fecal calprotectin, however, allowed the sensitivity to be raised to close to 100% [32]. The optimal combination of strategies to reduce diagnostic delay in CD needs further investigation.

Scarce data are available on UC. The type of symptoms, which are generally more alarming and disabling than in CD, and the high correlation between symptoms and intestinal inflammation might be associated with earlier diagnosis than in CD. The IMPACT survey showed that approximately 18% of UC patients had significant diagnostic delays (≥ 5 years) since the onset of symptoms [26], meaning that this may be an important issue for UC. At the moment, no clear data are available on the impacts of delayed diagnosis and delayed start of advanced therapies on the long-term course of UC. Further research is then needed.

## 9. Future Directions

Timely diagnosis and treatment may offer the opportunity to alter the natural history of IBD [18]. Earlier diagnosis may allow a window of opportunity to initiate disease-modifying therapy before irreversible bowel damage has occurred [16]. Consequently, this would lead to an improvement in the quality of life and a reduction in healthcare and social costs [35,36].

There are still some main challenges to face in the near future. Firstly, symptoms at the first presentation of IBD are non-specific and may be difficult to interpret. Moreover, one-third of patients will not have any or will have very mild symptoms. The use of the Red Flags Index together with non-invasive tests, like fecal calprotectin, may help GPs or non-IBD specialists to identify patients with high suspicion of IBD and to correctly refer these patients to referral specialists/units [31,32]. Data on the use of this combined strategy in large populations in different countries or regions are lacking. Secondly, the data indirectly show that awareness of IBD in the general population seems to be very low, resulting in underestimating or ignoring possible early symptoms or signs of IBD, or even reducing the chance that patients complete their diagnostic workup [32]. This aspect needs further investigation. Efforts should be made to provide continuous education not only directed to healthcare professionals or patients with established diagnoses, but especially directed to the general population, as is usually undertaken for cancer, diabetes, cardiovascular diseases, and even genetic diseases. If subjects know that some symptoms may reveal underlying and undiagnosed IBD, and they know the possible implications of delayed diagnosis, this may help anybody with red flags to see a doctor at the appropriate time. Educational programs, screening campaigns, and other similar initiatives directed to the general population may be helpful, although no data are available on their effectiveness. Besides the availability of non-invasive tools for healthcare professionals, educational campaigns may enhance public awareness of IBD, facilitating early connection between a potential patient and a GP or specialist. Collaboration between stakeholders, including patients’ associations, may be crucial in this context to spread awareness and knowledge about IBD in the general population.

Finally, data on the long-term impact of early diagnosis and referral in large multicenter populations of IBD patients are lacking, as well as a comparison of long-term effects in CD compared with UC. All these aspects need further research.

## 10. Conclusions

Diagnostic delay significantly impacts the natural history of IBD by increasing the risk of worse long-term outcomes. The combination of the Red Flags Index calculation and fecal calprotectin may help reduce the time to diagnosis and allow patients to start effective treatments within the window of opportunity.

## Figures and Tables

**Table 1:** Diagnostic delay and outcomes of Crohn’s disease.

Author	Diagnostic Delay	Outcome	Ratio	*p*-Value
Schoepfer et al. [7]	4–9 months	Bowel stricture	OR 1.55	0.047
	≥25 months		OR 1.76	0.011
Pellino et al. [6]	>18 months	CD-related surgery	NA	0.02
Schoepfer et al. [21]	4–9 months	CD-related surgery	OR 1.527	0.056
Moon et al. [22]	10–24 months	Perianal fistula	OR 1.572	0.032
Li et al. [5]	≥25 months	CD-related surgery	OR 1.895	0.004

**Table 2:** The Red Flags Index to screen patients with suspected Crohn’s disease [31].

Parameter	Multiplier
Non-healing or complex perianal fistula or abscess or perianal lesions	5
First-degree relative with confirmed inflammatory bowel disease	4
Weight loss (5% of usual body weight) in the last 3 months	3
Chronic abdominal pain (>3 months)	3
Nocturnal diarrhea	3
Mild fever in the last 3 months	2
No abdominal pain 30–45 min after meals	2
No rectal urgency	2

Every parameter is scored 0 if absent or 1 if present, and then it is multiplied by the relevant multiplier. The sum of each sub-score results in the final score. A score of ≥8 identifies a subject with a high probability of having underlying Crohn’s disease.

## Data Availability

Not applicable.
